# Enrichment of Polychlorinated Biphenyls from Aqueous Solutions Using Fe_3_O_4_ Grafted Multiwalled Carbon Nanotubes with Poly Dimethyl Diallyl Ammonium Chloride

**DOI:** 10.3390/ijms13056382

**Published:** 2012-05-23

**Authors:** Shaolin Zeng, Yuting Cao, Weiguo Sang, Tianhua Li, Ning Gan, Lei Zheng

**Affiliations:** 1 The State Key Laboratory Base of Novel Functional Materials and Preparation Science, Faculty of Material Science and Chemical Engineering, Ningbo University, Ningbo 315211, China; E-Mails: zengshaolin7320@126.com (S.Z.); caoyuting@nbu.edu.cn (Y.C.); sangweiguo@nbu.edu.cn (W.S.); litianhua@nbu.edu.cn (T.L.); 2 Clinical Laboratory Center, Nanfang Hospital, Southern Medical University, Guangzhou 510515, China

**Keywords:** Fe_3_O_4_ nanoparticles, MWCNTs, solid-phase extraction, polychorinated biphenyls, trace levels

## Abstract

In this paper, Fe_3_O_4_ nanoparticles (Fe_3_O_4_ NPs) grafted carboxyl groups of multiwalled carbon nanotubes with cationic polyelectrolyte poly (dimethyldiallylammonium chloride) (PDDA) (MWCNTs-COO^−^/PDDA@Fe_3_O_4_), are successfully synthesized and used for the extraction of six kinds of major toxic polychorinated biphenyls (PCBs) from a large volume of water solution. The hydrophilicity of the PDDA cage can enhance the dispersibility of sorbents in water samples, and the superparamagnetism of the Fe_3_O_4_ NPs facilitate magnetic separation which directly led to the simplification of the extraction procedure. With the magnetic solid-phase extraction (MSPE) technique based on the MWCNTs-COO^−^/PDDA@Fe_3_O_4_ sorbents, it requires only 30 min to extract trace levels of PCBs from 500 mL water samples. When the eluate condensed to 1.0 mL, concentration factors for PCBs became over 500. The spiked recoveries of several real water samples for PCBs were in the range of 73.3–98.9% with relative standard deviations varying from 3.8% to 9.4%, reflecting good accuracy of the method. Therefore, preconcentration of trace level of PCBs by using this MWCNTs-COO^−^/PDDA@Fe_3_O_4_ sorbent, which are stable for multiple reuses, from water solution can be performed.

## 1. Introduction

Polychlorinated biphenyls (PCBs), a class of chlorinated aromatic hydrocarbon chemicals that are now known as a part of the persistent organic pollutants (POPs), are highly toxic and resistant to degradation in the environment [1]. Although the marketing, manufacture, and application of PCBs have been banned for many years in most countries, concerns over these chemicals are never suspended because of their accumulation in the animal food chain on a nearly global scale. So the effective enrichment and identification of lowly concentrated PCBs in the environment is attracting a great deal of research attention due to human health concerns [2–6]. Adsorption, as one of the most cost-effective technologies, always acts as a method for researchers to study for the removal of organic pollutants. The understanding of the sorption behaviors of PCBs on different adsorbents is important for the enrichment of PCBs in aqueous solution contaminated with PCBs. Therefore, the novel adsorbents with high adsorption capacity are important for the detection of PCBs in the environmental pollution clean up.

Carbon nanotubes (CNTs) are relatively new adsorbents which have been proven to possess excellent adsorption capacity for aromatic hydrocarbons compounds [7–9]. The results suggest that CNTs are a very suitable material in the removal of aromatic hydrocarbons pollutants from large volumes of aqueous solutions. However, the inherent insolubility of CNTs in most organic and aqueous solvents limits the manipulation of CNTs in solution phase, which greatly hinders the application of CNTs in reality. Poly (dimethyldiallylammonium chloride) (PDDA) is a water-soluble, quaternary ammonium, cationic polyelectrolyte polymeric materials when dissolved in aqueous solutions usually acts as a positively charged colloid [10,11] and can be easily coated on the negatively charged surface of the MWCNTs by electrostatic interactions. The polymeric materials can also remove PCBs effectively through the formation of inclusion complexes [12]. If the PDDA molecules are grafted on the surfaces of MWCNTs, the solubility and extraction efficiency of the composite materials can both be enhanced.

In recent years, many researchers have studied the modification of CNTs by introducing special functional groups on its surface in order to enhance its adsorption capacity for organic aromatic compounds and improve its dispersion property in aqueous solutions [13–15]. These nanosized sorbents have great solubility and high extraction efficiency. However, these sorbents have to be separated from the liquid phase by centrifugation, and since nanomaterials which makes it more difficult to be separated in aqueous small particle size. In order to overcome such disadvantages, our groups have developed a novel solid-phase extraction (SPE) method based on mixed ionic surfactants modified MWCNTs, where magnetic nanoparticles are adsorbed on the ionic surfactants to form MWCNTs-COO^−^/PDDA@Fe_3_O_4_. Compared with traditional SPE sorbents [16–19], the magnetic property endues the sorbents with magnetic separation ability so that the time-consuming SPE steps can be saved. In our experiment, the regenerability and reusability of these MNPs for PCBs removal from aqueous solution will be specially studied and proofed.

Cheng *et al.* use carbon nanotubes/Fe_3_O_4_ with PDDA developed a tyrosinase (Tyr) biosensorand which can test electric signal by CNTs’ electroconductibility, to detect the concentration of coliforms [20]. However, there are obvious differences for the magnetic material’s application and mode of action between their article and ours. In our work, we use carbon nanotubes/Fe_3_O_4_ with PDDA to adsorb PCBs through π–π absorption mechanism in water samples. Our study aims to develop a novel magnetic nanoparticle of MWCNTs-COO^−^/PDDA@Fe_3_O_4_ as stationary phase of SPE and their potential applications in the enrichment of PCBs from environmental water samples. To examine the feasibility of this approach, we select six kinds of major toxic PCBs (2,4,4′-Trichlorobiphenyl(PCB 28), 2,2′,5,5′-tetrachlorobiphenyl(PCB 52), 2,2′,4,5,5′-pentachlorobiphenyl (PCB 101), 2,2′,3,4,4′,5-hexachlorobiphenyl (PCB 138), 2,2′,4,4′,5,5′-hexachlorobiphenyl (PCB 153) and 2,2′,3,4,4′,5,5′-heptachlorobiphenyl (PCB 180)) as model compounds. To combine SPE technique with gas chromatography-mass spectrometry (GC-MS) detection, a highly efficient and fast SPE-GC/MS analytical method was established. Moreover, the performances of PCBs analysis were compared among MWCNTs-SPE, MWCNTs-COO^−^/PDDA-SPE, MWCNTs-COO^−^/PDDA@Fe_3_O_4_-SPE and C18-NH_2_-SPE methods.

## 2. Results and Discussion

### 2.1. Characterization of Sorbents

#### 2.1.1. Particle Size and Morphology

**Figure 1:**
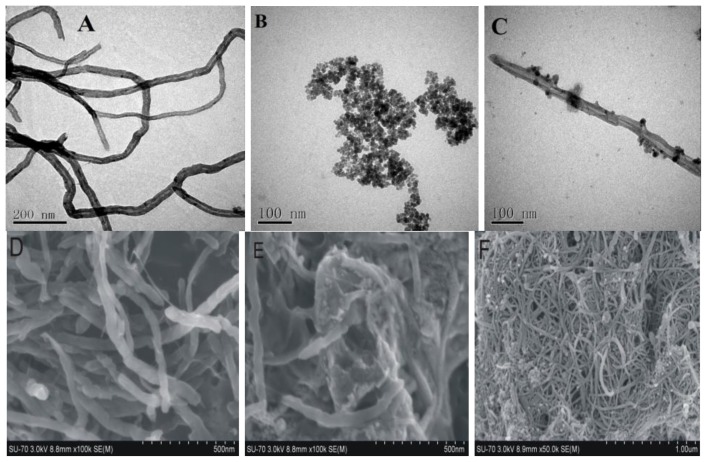
Transmission electron microscope (TEM) images of (**A**) Multi-walled carbon nanotubes (MWCNTs), (**B**) Fe_3_O_4_ and (**C**) MWCNTs-COO^−^/PDDA@Fe_3_O_4_; and Scanning electron microanalysis (SEM) images of (**D**) MWCNTs and MWCNTs-COO^−^/PDDA@Fe_3_O_4_ under (**E**) 500 nm and (**F**) 1.00 μm.

By SEM and TEM images (Figure 1), the microstructure transformations and difference of MWCNTs, Fe_3_O_4_ and MWCNTs-COO^−^/PDDA@Fe_3_O_4_ can be observed. In Figure 1A, the purified MWCNTs show smooth and uniform surface morphology. Figure 1B shows the TEM images of (Fe_3_O_4_ NPs). Fe_3_O_4_ NPs appear quasi-spherical in shape with an average diameter of about 20 nm. After MWCNTs functioned with PDDA, the negatively charged Fe_3_O_4_ NPs can easily assemble on the surface of MWCNTs through electrostatic interactions. Figure 1C displays that numerous Fe_3_O_4_ NPs are successfully absorbed onto the surface of the MWCNTs. Figure 1D shows the SEM images of MWCNTs. From Figure 1E–F, we can observe that numerous Fe_3_O_4_ NPs are successfully absorbed onto the surface of the MWCNTs by SEM under different sizes. The results show that the surfaces of the MWCNTs before Fe_3_O_4_ grafting treatment are smooth and tidy.

#### 2.1.2. IR Spectra

**Figure 2:**
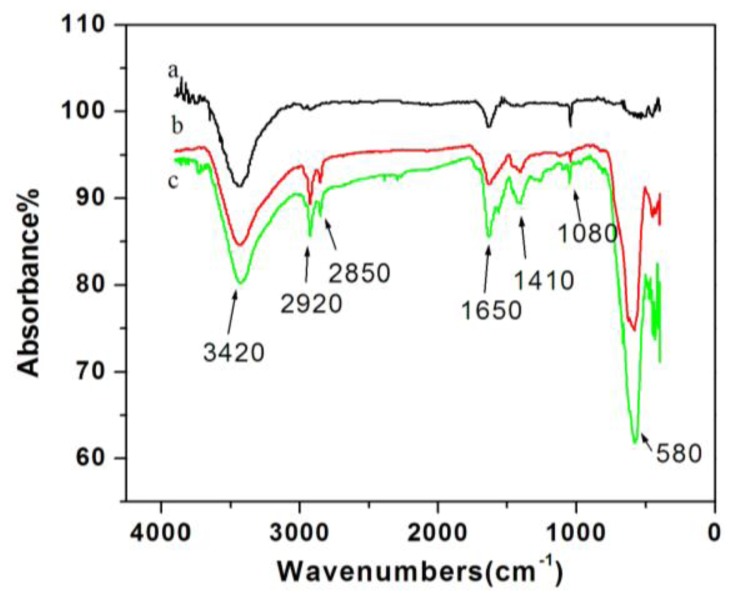
IR spectra of (**a**) MWCNTs -COO^−^/PDDA; (**b**) Fe_3_O_4_; (**c**) MWCNTs-COO^−^/PDDA@Fe_3_O_4_.

Figure 2 displays IR spectra of Fe_3_O_4_, MWCNTs-COO^−^/PDDA, and MWCNTs-COO^−^/PDDA@Fe_3_O_4_. The stretching vibrations of hydroxyl groups (OH) usually form a broad band region at 3200–3650 cm^−1^ [21]. Thus, the peak at 3420 cm^−1^ can be assigned to the overlap of the band of Fe_3_O_4_ nanoparticles, the band of oxidized MWCNTs with PDDA and MWCNTs-COO^−^/PDDA@Fe_3_O_4_ nanocomposites. As shown in Figure 2, after Fe_3_O_4_ nanoparticles function with oleic acid, the stretching vibration of hydroxyl groups disappears, while a C–H stretching vibrating band at 2920 and 2850 cm^−1^ arises, and 1650 cm^−1^ absorption peaks become C=O. The peak at 1080 cm^−1^ can be assigned to C–O vibration in Fe_3_O_4_ nanoparticles and in oxidized MWCNTs. This indicates that the carboxyl is successfully attached to the magnetic nanoparticles by reactions with the hydroxyl groups on the surface of Fe_3_O_4_. Once Fe_3_O_4_ nanospheres are combines with the surface of MWCNTs-COO^−^/PDDA compound, the hydroxyl group absorption and Fe_3_O_4_ nanospheres absorption can be greatly enhanced, and C–H stretching vibrating band at 2920 and 2850 cm^−1^ appears again. These groups are caused by the Fe_3_O_4_ nanospheres, indicating successful function oleic acidification of Fe_3_O_4_ nanospheres on the surface of the MWCNTs -COO^−^/PDDA nanocomposites.

#### 2.1.3. Magnetic Properties

**Figure 3:**
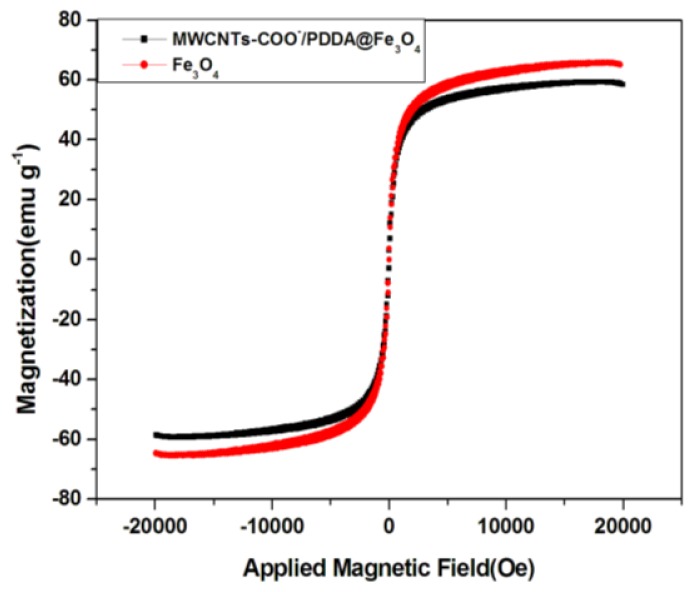
Vibrating sample magnetometer (VSM) supermagnetization curves of Fe_3_O_4_ and MWCNTs-COO^−^/PDDA@Fe_3_O_4_.

The magnetic property of the MWCNTs-COO^−^/PDDA@Fe_3_O_4_ nanocomposite is investigated by VSM. Figure 3 shows the supermagnetization curves of Fe_3_O_4_ and MWCNTs-COO^−^/PDDA@Fe_3_O_4_ at room temperature. Maximum saturation supermagnetizations of Fe_3_O_4_ and MWCNTs-COO^−^/PDDA@Fe_3_O_4_ are measured at 65.42 and 59.54 emu·g^−1^, respectively. Although the addition of nonmagnetic portion leads to a decreased saturation supermagnetizations, the obtained MWCNTs-COO^−^/PDDA@Fe_3_O_4_ still have a high saturation supermagnetization of 59.54 emu·g^−1^. According to Ma’s study, a saturation supermagnetization of 16.3 emu·g^−1^ is enough for magnetic separation from solution with a magnet [22]. These imply that MWCNTs-COO^−^/PDDA@Fe_3_O_4_ sorbents can be dispersed into water solution readily and the magnetic sorbent loaded with analytes can be isolated from the matrix conveniently by using an external magnet when necessary. Once the external magnetic field is taken away, these sorbents can redisperse rapidly.

#### 2.1.4. XRD

**Figure 4:**
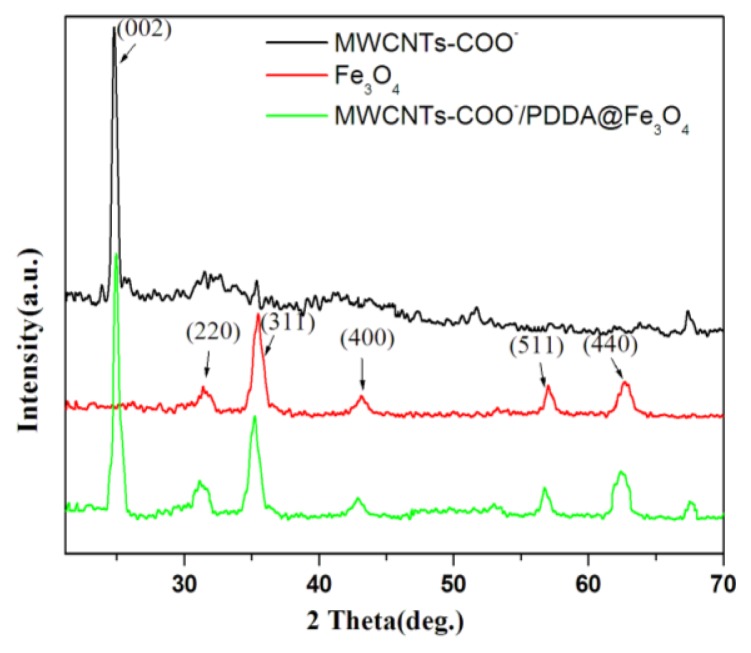
X-ray diffraction (XRD) patterns of MWCNTs-COO^−^, Fe_3_O_4_ and MWCNTs- COO^−^/PDDA@ Fe_3_O_4_.

The XRD pattern of the MWCNTs-COO^−^, Fe_3_O_4_ and MWCNTs-COO^−^/PDDA@Fe_3_O_4_ are presented in Figure 4. The appearance of diffraction peak at 2*θ* = 26° (002) could be ascribed to the reflection of the MWCNTs. Seven diffraction lines are observed in the representative XRD pattern of Fe_3_O_4_ at 2*θ* = 30.1°, 35.4°, 43.3°, 57.3° and 62.8°. These diffraction lines can be assigned to the (220), (311), (400), (422), (511) and (440) reflections, respectively, of the pure cubic spinel crystal structure of Fe_3_O_4_ with cell constant *α* = 8.397 Å (JCPDS card No.19-0629). From the XRD pattern of MWCNTs-COO^−^/PDDA@Fe_3_O_4_ analysis, the main characteristic peaks of Fe_3_O_4_ and MWCNTs located at 26°, 30.1°, 35.4°, 43.3°, 57.3°and 62.8°, indicating that the product is composed of two phases: Fe_3_O_4_ and MWCNTs.

### 2.2. Optimization of Extraction Conditions

#### 2.2.1. Magnetic Separation and Adsorption Equilibrium Time

**Figure 5:**
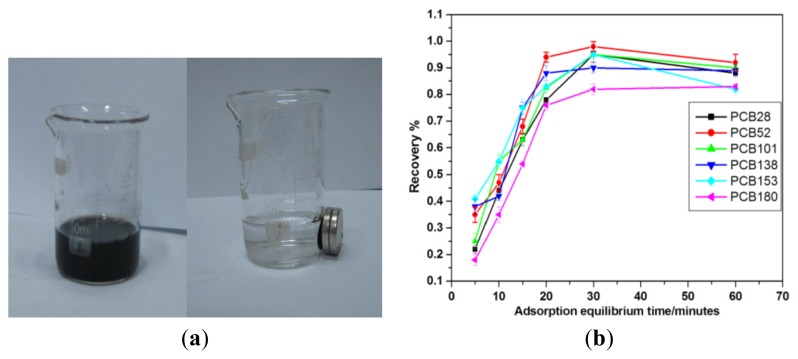
(**a**) Dispersion and magnetic separation of the sorbent; (**b**) Equilibrium time.

The MWCNTs-COO^−^/PDDA@Fe_3_O_4_ sorbents possess superparamagnetism and large saturation magnetization, which makes the sorbents completely isolated from solution within only about 2 min by a strong Nd-Fe-B magnet (Figure 5a). To get a homogeneous suspension, the MNPs sorbent is dispersed in water solution by ultrasonic treatment. To reach adsorption equilibrium, a certain standing time is required after the sorbents are dispersed into the solution. As shown in Figure 5b, the recoveries of 6 kinds of PCBs are all enhanced with increasing time, and the maximum recoveries of these analytes are obtained when the standing time increases to 20–30 min. The short diffusion route of nanosized sorbents results in rapid extraction dynamics. From the Figure 5b, 30 min is sufficient for 0.05 g MWCNTs-COO^−^/PDDA@Fe_3_O_4_ sorbent to extract 80% of each target from 500 mL of water solution.

#### 2.2.2. Effect of the Solution pH

**Figure 6:**
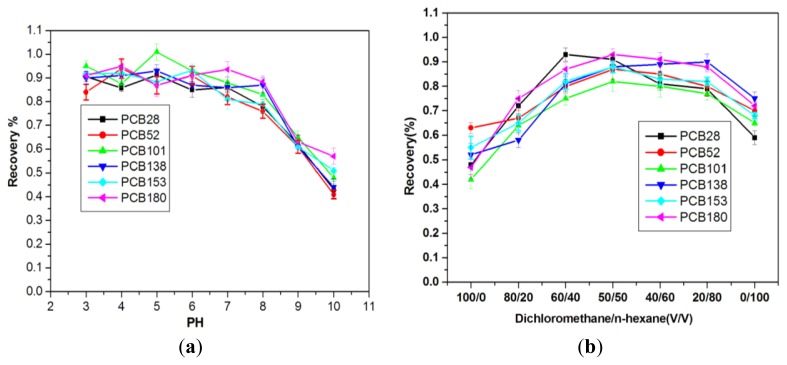
(**a**) Effects of pH values on the recoveries of six PCBs; (**b**) Effects of solvent type on the recoveries of six PCBs.

Solution pH would change the charge property of the MWCNTs-COO^−^/PDDA@Fe_3_O_4_ sorbent, and thus influence the adsorption of sorbent to targets. In this work, the effect of solution pH on the extraction of targets is investigated in the pH range of 3.0–10.0. As can be seen from Figure 6a, the sorption percentage of PCBs on MWCNTs-COO^−^/PDDA@Fe_3_O_4_ fluctuates very little in pH range of 3–8, which suggests that MWCNTs-COO^−^/PDDA@Fe_3_O_4_ are excellent adsorbents for PCBs’ removal from large volumes of aqueous solutions. When the pH > 8, the sorption percentage of PCBs on MWCNTs-COO^−^/PDDA@Fe_3_O_4_ decreases with increasing pH values. This can be ascribed to the fact that more oxygen containing groups (such as –COOH and –OH) on MWCNTs-COO^−^/PDDA@Fe_3_O_4_ surfaces are ionized (carrying negative charge) at high pH values, more water molecules are adsorbed to prevent PCBs which are closed to the adsorbent. The formation of a water cluster on oxygen-containing groups blocks the access of PCBs to the adsorption sites of MWCNTs-COO^−^/PDDA@Fe_3_O_4_ and thereby results in less adsorption of PCBs. Lu *et al.* [23] reported that the adsorption of trihalomethanes on MWCNTs fluctuate very little in pH ranging from 3 to 7, and decrease with increasing pH over 7. Peng *et al.* [24] reported that the sorption of 1, 2-dichlorobenzene from aqueous solution to CNTs remaines almost constant in the pH range 3–10. As seen from our experiment, the pH independent adsorption of PCBs on MWCNTs-COO^−^/PDDA@Fe_3_O_4_ at pH < 8 is quite important for the application of MWCNTs-COO^−^/PDDA@Fe_3_O_4_ in PCB pollution cleanup in real work.

#### 2.2.3. Effect of Elution Solvent

The most commonly used solvents are dichloromethane and n-hexane in PCBs analysis. In this study, we also apply these solvents to elute PCBs from MWCNTs-COO^−^/PDDA@Fe_3_O_4_-SPE syringe. Moreover, the recoveries of PCBs compare among dichloromethane, *n*-hexane, dichloromethane/*n*-hexane (80:20, *v*/*v*), dichloromethane/*n*-hexane (50:50, *v*/*v*) and dichloromethane/*n*-hexane (20:80, *v*/*v*) which were loaded at 2.5 mL on the syringe. As the results shown in Figure 6b, different solvents possess different elution efficiency. The recoveries of most PCBs congeners eluted by dichloromethane are less than 50%. The performance of *n*-hexane is better than dichloromethane, showing an average recovery exceeding 57% for *n*-hexane. *n*-Hexane is known as a non-polar solvent and dichloromethane is a polar solvent with a polarity index of 3.1. In fact, the mixture of *n*-hexane and dichloromethane is often used to elute PCBs from silica gel column due to its proper polarity index. Three mixtures of *n*-hexane and dichloromethane all result in good recoveries of PCBs congeners, an average of near 80% for both mixtures of n-hexane and dichloromethane. However, the performance of *n*-hexane/dichloromethane (50:50) is better and more stable than that of *n*-hexane/dichloromethane (40:60) and *n*-hexane/dichloromethane (60:40). Therefore, the mixture of *n*-hexane/dichloromethane (50:50) becomes the solvent to elute MWCNTs-COO^−^/PDDA@Fe_3_O_4_-SPE syringe in the following tests.

#### 2.2.4. Volume of Elution Solvent

**Figure 7:**
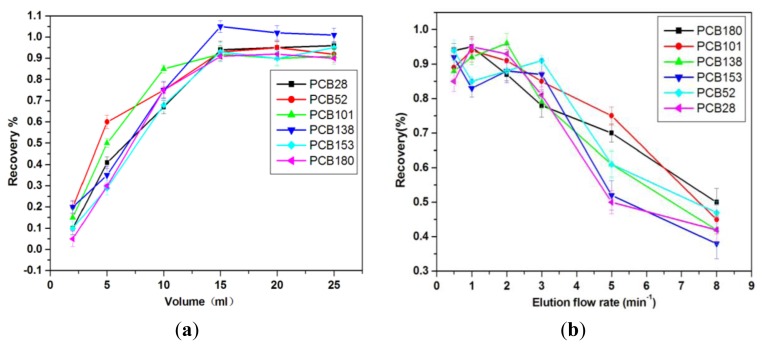
(**a**) Effects of Volume on the recoveries 6 PCBs; (**b**) Effects of Elution flow rate on the recoveries of six PCBs.

To study the effect of desorption volume, desorption solvent from 2 mL to 25 mL is used to elute the analytes after sample extraction and washing step. From the Figure 7a, most proportion of each congener is eluted by 15 mL of *n*-hexane/dichloromethane (50:50), and the recoveries of PCBs congeners all exceeded 88% at 15 mL. After Elution, volume becomes higher than 15 mL, recovery rates do not increase; they even occasionally decrease. In addition, less than 15 mL of *n*-hexane/dichloromethane (50:50, *v*/*v*) mixture cannot elute the extracted PCBs from the MWCNTs-COO^−^/PDDA@Fe_3_O_4_-SPE syringe totally. Therefore, 15 mL of n-hexane/dichloromethane (50:50) become the best choice to elute PCBs from MWCNTs-COO^−^/PDDA@Fe_3_O_4_-SPE syringe in the following tests.

#### 2.2.5. Effect of Desorption Flow Rate

The desorption flow rate is optimized in the range of 0.5–8 mL·min^−1^. The high flow rate may cause high back pressure which will not favor the operation of extraction. Meanwhile, too low flow rate may prolong the whole analysis time. In the Figure 7b, although the recovery rate of the desorption flow rate of 0.5 mL and 1 mL are relatively high, the elution time becomes quite long. When the flow rates go over than 2 mL·min^−1^, the recovery rates of the PCBs begin to decline. This is probably because that the speed of flow rate is too fast, while the PCBs still partly retain on the absorbent, causing the recovery rate declined. Therefore, a flow rate of 2 mL·min^−1^ is chosen to obtain satisfactory desorption efficiency within a short time.

### 2.3. Reusability of the Sorbents

**Figure 8:**
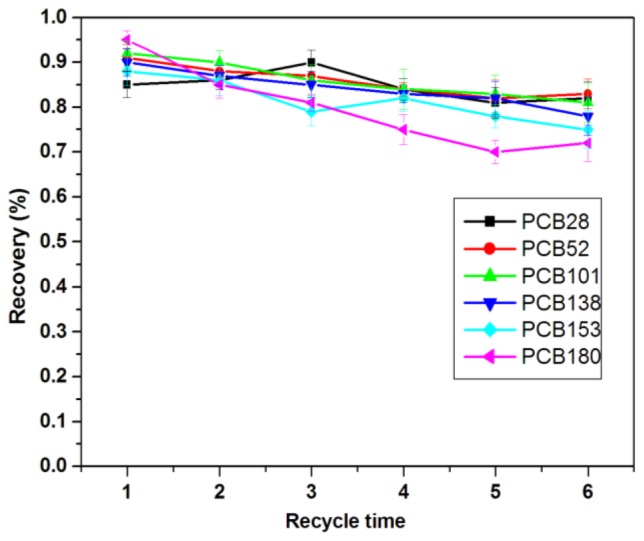
Effects of recycle time on the recoveries of six PCBs.

In order to investigate the recycling of the nanoparticle sorbents, the MWCNTs-COO^−^/PDDA@Fe_3_O_4_ sorbents used in SPE procedure are washed by 5 mL of acetonitrile before applied in the next SPE procedure. The recoveries of PCBs are listed in Figure 8. The sorbents can be reused six times without much sacrifice of the recoveries of analytes. As the number of cycles increases, Fe_3_O_4_ gradually fall off the sorbents, which results in the gradual disappearance of the magnetism of the sorbents.

### 2.4. Method Validation

A series of standard solutions (10, 20, 40, 60, 100, 200 ng·mL^−1^) are prepared to obtain the linear range of each PCB congener. From the Table 1, the linearity range of standard solutions shows between 10~200 ng·mL^−1^. Linear correlation coefficients (*r*^2^) of these congeners vary between 0.9985 and 0.9998. Limits of detection (LODs), defined as signal to noise ratio of 3:1, ranges from 0.031 to 0.069 ng·mL^−1^ for the PCBs congeners. The limits of quantification (LOQs) based upon a signal-to-noise ratio of 10:1 for PCB 28, PCB 52, PCB 101, PCB 153, PCB 138 and PCB 180 are 0.1, 0.17, 0.23, 0.19, 0.14 and 0.16 ng·mL^−1^, respectively. Double distilled water spiked with six PCBs at 10 ng·mL^−1^ and 1 ng·mL^−1^ are used for the precision study. The relative standard deviations (R.S.D., *n* = 5) of PCBs with low concentration range from 5.8% to 9.4%. And the relative standard deviations (R.S.D., *n* = 5) of PCBs with high concentration ranged from 3.5% to 6.7%.

### 2.5. Determination of PCBs in Environmental Water Samples

To test the MWCNTs-COO^−^/PDDA@Fe_3_O_4_-SPE method, this paper analyzes tap water, school water, and river water samples. As shown in Figure 9, the results indicate that school water was contaminated by PCB 28 and PCB 52.

Table 2 summarizes the recoveries and concentrations of the target compounds in these real water samples, expressed as mean value (*n* = 3). Among three environmental water samples, six PCBs are detected and the concentrations are relatively high. Meanwhile trace levels of PCB 28 and PCB 52 are found in the school water sample and river water sample. The results show that the recoveries of spiked environmental water samples are very satisfactory. The recoveries of PCBs range from 73.3% to 98.9%.

### 2.6. Comparison of MWCNTs-COO-/PDDA@Fe_3_O_4_-SPE with Other Extraction Methods of PCBs

In the same concentration factor cases, Table 3 summarized the LODs, relative recovery (RR) and analysis time and comparison of MWCNTs-SPE, MWCNTs-COO^−^/PDDA-SPE, MWCNTs-COO^−^/PDDA@Fe_3_O_4_-SPE and C18-SPE, the extraction and determination of PCBs in water samples, expressed as mean value (*n* = 3). In the experiment, we employ all the materials except MWCNTs-COO^−^/PDDA@Fe_3_O_4_ as stationary phase of SPE to enrich and purify PCBs in aqueous solutions. The recoveries of PCB congeners range from 68.2% to 76.8%, 78.4% to 98.6%, 78.1% to 98.3% and 75.9% to 105.4% for MWCNTs-SPE, MWCNTs-COO^−^/PDDA-SPE, MWCNTs-COO^−^/PDDA@Fe_3_O_4_-SPE and C18-NH_2_-SPE methods, respectively. We find that when the PDDA molecules are grafted on the surfaces of MWCNTs, the extraction efficiency of the composite materials is enhanced. From Table 3, we can see that the method of MWCNTs-COO^−^/PDDA@Fe_3_O_4_-SPE is the most time-saving with its simple operation, which only took 2.4 h. The results also show MWCNTs-COO^−^/PDDA@Fe_3_O_4_-SPE is an efficient and simple method to analyze PCBs in sewage water samples.

## 3. Experimental Section

### 3.1. Chemicals and Materials

PCB 28, PCB 52, PCB 101, PCB 138, PCB 153 and PCB 180 obtained from Accu Standard (New Haven, CT, USA). Poly (diallyl dimethyl ammonium chloride) (PDDA, 20%, *w*/*w*, in water, MW = 100000–200000) purchased from Sigma-Aldrich. The carboxyl of multi-walled carbon nanotubes (MWCNTs-COOH, purity > 95%, diameter < 5 nm, length 0.5–15 μm) were purchased from Nanoport. Co. Ltd. (Shenzhen, China). Ferric chloride (FeCl_3_·6H_2_O), ferrous chloride (FeCl_2_·4H_2_O), Oleic acid, ammonia, *n*-hexane and dichloromethane were purchased from Beijing Chemicals Corporation (Beijing, China). The syringe filters was purchased from Xingya (Shanghai). All reagents used in the experiment were of analytical reagent grade and used without further purification.

### 3.2. Preparation of Fe_3_O_4_ Nanospheres

The preparation of magnetic nanoparticles was based on the previously reported procedures [25]. Firstly, 4.3 g FeCl_2_·4H_2_O and 11.6 g FeCl_3_·6H_2_O were dissolved in 350 mL deionized water and heated to 80 °C under the N_2_ atmosphere with vigorous stirring. Then, 20 mL of 25 wt% NH_4_OH was rapidly added into the above-prepared solution and vigorously stirred for 5 min. Finally, 1 mL oleic acid was added into the suspension and allowed to react for 25 min to form the tar-like black magnetic gel precipitate. The obtained magnetic particles were sequentially washed with deionized water and ethanol to remove the excess oleic acid, and then dried in vacuum for use. 160 mL concentration of 10 mg mL^−^^1^ of dissolved KMnO_4_ was added after fluid ultrasonic cleaning 8 h in the ultrasonic instrument. After magnetic separation, the precipitate was washed with deionized water three times. Finally, the powder was dried at 60 °C under a vacuum, in preparation for application.

### 3.3. Synthesis of MWCNTs-COO^−^/PDDA@Fe_3_O_4_

MCNTs-COOH was purified by ultrasonication in 0.5 M HCl for 4 h. The products were washed with water and dried at 50 °C overnight [25]. Fifty milligrams of purified MCNTs-COOH (MWCNTs-COO^−^) was dispersed into a sodium chloride aqueous solution (0.5 mol·L^−1^, 114 mL). Then the PDDA solution (20%, 6 mL) was dropped into the mixture under sonication and vigorous stirring for 30 min. The mixture was centrifuged and washed with deionized water for several times to remove the residual PDDA solution. Then 60.0 mg Fe_3_O_4_ nanospheres powder was added to the mixture and the solution (50 mL) was vigorously stirred for 1 h. The resulting MWCNTs-COO^−^/PDDA@Fe_3_O_4_ was thoroughly washed with deionized water, collected by magnetic separation, and dried at 40 °C under vacuum for 12 h. Figure 9 schematically illustrated the synthesis process of MWCNTs-COO^−^/PDDA@Fe_3_O_4_.

### 3.4. Material Characterization

The particle size and structure of the sorbents were observed by using a Hitachi 8100 transmission electron microscope (TEM, Japan) and Hitachi SU-70 scanning electron microanalysis (SEM, Japan). The infrared (IR) spectra of the obtained sorbents were taken in KBr pressed pellets on a NEXUS 670 infrared Fourier transform spectrometer (Nicolet Thermo, Waltham, MA). Magnetic property was analyzed using a vibrating sample magnetometer (VSM, LDJ9600). The XRD characterization was performed using X-ray diffraction (Bruker, D8 Focus) with Cu Kα radiation at room temperature.

### 3.5. Magnetic SPE Procedure

Standard stock solutions (100 μg·L^−1^) containing 6 kinds of PCBs were prepared in *n*-hexane and stored at 0 °C. All the working solutions of PCBs were 200 ng·L^−1^ and prepared daily by appropriate dilution of the stock solutions with ultra-pure water. The adsorption experiments were carried out in beakers at room temperature. Firstly, 0.05 g of the MWCNTs-COO^−^/PDDA@Fe_3_O_4_ sorbents were rinsed and activated in 1mL of methanol, and then dispersed into a 500 mL working solutions. The mixture was sonicated for 5 min and stirred for 30 min. Subsequently, an Nd-Fe-B strong magnet was deposited at the bottom of the beaker and the sorbents were isolated from the solution. After about 30s, the solution became clear and the supernatant was decanted.

**Figure 10:**
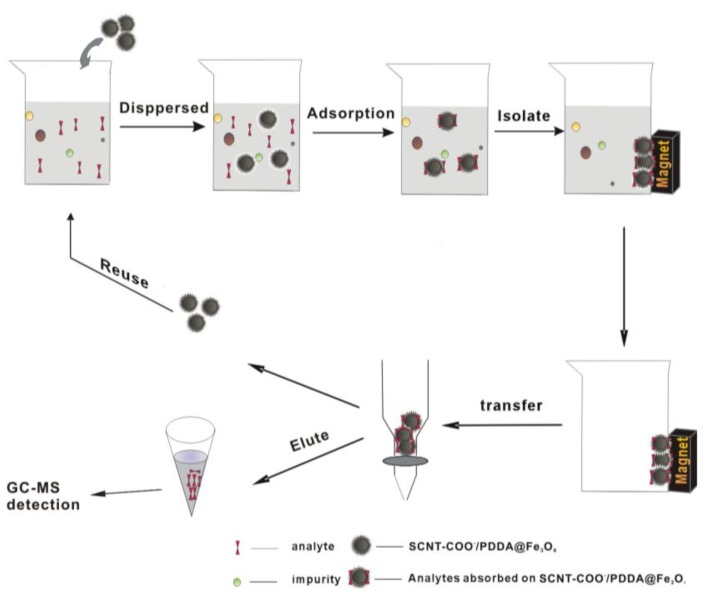
Adsorption process.

For the desorption step, firstly, the pre-concentrated analytes were transferred to the syringes of organic membrane (0.45 μm), drying at 40 °C under vacuum for 1 h. Then the syringes were eluted from the isolated sorbents with *n*-hexane and methylene chloride. Secondly, 15 mL mixture of dichloromethane and *n*-hexane (1:1, *v*/*v*) was injected into the the syringe with infusion pump at 2 mL·min^−1^ and the eluate was collected with the 20 mL centrifugal tubs. Thirdly, the eluate was dried with a stream of nitrogen at 40 °C and the residue was dissolved in 1 mL of *n*-hexane with concentrated sulfuric acid to further acidification. Finally, 1 μL of the supernate was injected rapidly into the gas chromatograph for the analysis by GC-MS. The sdsorption process is shown in Figure 10:

### 3.6. GC-MS Analysis

The GC-MS analysis was performed on SHIMADZU QP2010E GC-MS system equipped with a DB-5 MS column (30 m × 0.25 mm I.D. × 0.25 μm film thickness). The temperatures of injector and ion source were 280 °C and 220 °C, respectively. One microliter sample was injected by a manual in split stream mode. The carrier gas was helium at 0.7 mL·min^−1^. The oven temperature was programmed as follows: initial temperature 110 °C for 3 min, heated to 230 °C at 20 °C·min^−1^and held for 5 min, then ramped at 5 °C·min^−1^ to 250 °C and held for 2 min, finally ramped at 20 °C·min^−1^ to 290 °C; EI (70 eV); ion source temperature 220 °C; scan range 45~650 amu·s^−1^; solvent delay time 7 min. Selected ion monitoring (SIM) was used to quantify the analytes.

### 3.7. Real Sample Preparation

Water samples were collected in our campus and in September 2011, and tap water samples were taken from our laboratory in Jiangbei District (Ningbo). River water samples were acquired from Yongjiang River and brook waste water was obtained from Ningbo University (Jiangbei District, Ningbo). All samples were collected randomly and filtered through 0.45 μm membranes to remove suspended particles. The filtered water samples were analyzed within 24 h.

## 4. Conclusions

In this study, magnetic nanoparticles were successfully synthetized functionalized with MWCNTs groups to improve their hydrophilicity and dispersibility in aqueous solution. The obtained materials were developed as magnetic SPE sorbents to extract PCBs from large volumes of environmental water samples. Compared with previous SPE methods, this SPE method had following merits: (a) After adsorption of analytes from water samples, the sorbents can be collected conveniently and rapidly with a magnet for elution due to superparamagnetism of the materials, which avoided the time-consuming column passing or filtration operation; (b) Nanoparticle sorbents possessed high adsorption capacity and rapid adsorption rates, so a low amount of sorbents and short equilibrium time were required to extract analytes from large volumes of water samples; (c) The sorbent can be reused, making it cost saving, environmentally friendly and efficient. This study has not only proposed a rapid and convenient SPE method to extract organic compounds from large volumes of water samples, but also extended the scope of application of SPE based on MWCNTs functionalized magnetic nanoparticles.

## Figures and Tables

**Figure 9:**
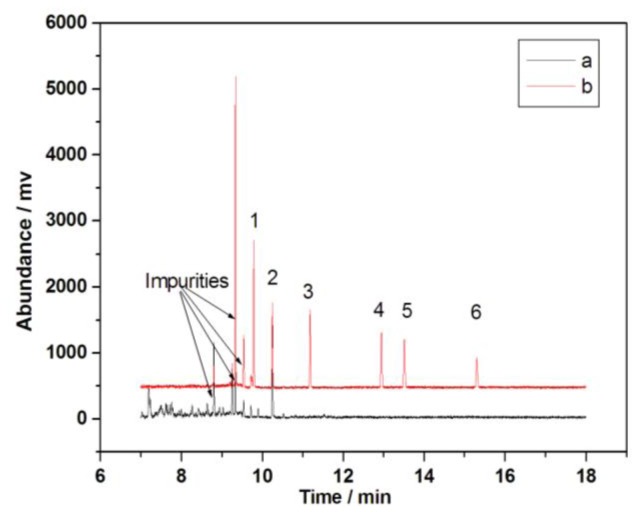
Chromatographs of school water (**a**) and school water samples spiked with 10 ng·mL^−1^ of PCBs (**b**). The samples were analyzed via GC-MS. Peak identification: 1.PCB 28, 2.PCB 52, 3.PCB 101, 4.PCB 153, 5.PCB 138, 6.PCB 180.

**Figure 9:**
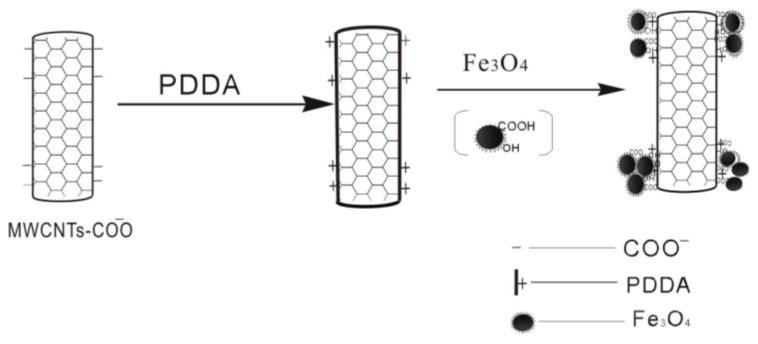
Synthesis strategy of MWCNTs-COO^−^/PDDA@Fe_3_O_4_ MNPs.

**Table 1:** The limits of detection, limits of quantification and reproducibility of the method.

Analytes	Calibration equations	Linear correlation coefficients (*r*^2^)	LOQ (ng·mL^−1^)	LOD (ng·mL^−1^)	RSD/%	

					Low	High
**PCB28**	*Y* = 110.715*x* + 24.516	0.9988	0.10	0.031	8.5	5.2
**PCB52**	*Y* = 70.586*x* + 16.165	0.9998	0.17	0.052	7.8	3.5
**PCB101**	*Y* = 25.675*x* + 18.382	0.9992	0.23	0.069	8.3	4.2
**PCB138**	*Y* = 36.707*x* + 22.908	0.9993	0.19	0.058	9.4	6.7
**PCB153**	*Y* = 31.704*x* + 13.181	0.9994	0.14	0.042	5.8	3.8
**PCB180**	*Y* = 20.413*x* − 15.181	0.9985	0.16	0.047	7.2	4.1

**Table 2:** Six kinds of major toxic recoveries of PCBs from water samples spiked with target analytes.

Analytes	Tap water			School water			River water		

	Real	Added	RR 1	Real	Added	RR 1	Real	Added	RR 1
					
	(ng·mL^−1^)	(ng·mL^−1^)	(%)	(ng·mL^−1^)	(ng·mL^−1^)	(%)	(ng·mL^−1^)	(ng·mL^−1^)	(%)
**PCB28**	ND 2	10	73.3%	2.7	10	71.2%	1.1	10	71.7%
		100	90.2%		100	88.2%		100	85.8%
**PCB52**	ND	10	79.8%	5.3	10	75.3%	0.8	10	78.3%
		100	94.8%		100	92.8%		100	93.4%
**PCB101**	ND	10	87.1%	ND	10	77.9%	ND	10	79.5%
		100	98.9%		100	91.9%		100	91.6%
**PCB138**	ND	10	90.2%	ND	10	89.2%	ND	10	88.7%
		100	95.7%		100	93.5%		100	96.3%
**PCB153**	ND	10	82.5%	ND	10	81.6%	ND	10	80.3%
		100	93.2%		100	96.7%		100	91.8%
**PCB180**	ND	10	78.1%	ND	10	79.1%	ND	10	81.1%
		100	95.7%		100	95.2%		100	95.7%

1 Relative recovery;

2 Not detected.

**Table 3:** Comparison of MWCNTs-COO^−^/PDDA@Fe_3_O_4_-SPE with other extraction methods for the determination of PCBs in water samples.

Methods	LODs (ng·mL^−1^)	RR (%)	Time of pretreatment (h)
MWCNTs-SPE	0.028–0.051	68.2–76.8	6.4
MWCNTs-COO^−^/PDDA-SPE	0.034–0.063	78.4–98.6	6.4
MWCNTs-COO^−^/PDDA@Fe_3_O_4_-SPE	0.027–0.059	78.1–98.3	2.4
C18-NH_2_-SPE	0.007–0.023	75.9–105.4	6.0
